# Relationship between parathyroid hormone levels and sarcopenia and nutritional status in patients undergoing hemodialysis

**DOI:** 10.1590/2175-8239-JBN-2025-0083en

**Published:** 2026-08-07

**Authors:** Claudia Campello Leal, Dejane de Almeida Melo, Alcione Miranda dos Santos, Maria Conceição Chaves de Lemos, Pedro Israel Lira, Claudia Porto Sabino Pinho

**Affiliations:** 1 Universidade Federal de Pernambuco, Departamento de Nutrição, Recife, PE, Brazil.; 2 Instituto de Gestão Estratégica de Saúde do Distrito Federal, Hospital de Base, Brasília, DF, Brazil.; 3 Universidade Federal do Maranhão, Recife, PE, Brazil.; 4 Universidade Federal de Pernambuco, Empresa Brasileira de Serviços Hospitalares, Hospital das Clínicas, Recife, PE, Brazil.

**Keywords:** Parathyroid Hormone, Renal Insufficiency, Chronic, Renal Dialysis, Sarcopenia, Malnutrition

## Abstract

**Introduction:**

The potential effect of elevated parathyroid hormone (PTH) levels on nutritional status and sarcopenia in chronic kidney disease (CKD) patients undergoing hemodialysis (HD) has not yet been well established.

**Objective:**

The aim of this study was to evaluate the status of sarcopenia and nutritional aspects according to PTH levels in HD patients.

**Methods:**

This cross-sectional study evaluated consecutive patients attending three HD clinics in Northeast Brazil. Patients were stratified based on PTH levels: (1) 0–199 pg/mL, (2) 200–599 pg/mL, and (3) ≥ 600 pg/mL. Group 1 was excluded due to older age, and groups 2 and 3 were matched for age, sex, and presence of diabetes. Sarcopenia was defined according to the European consensus criteria, assessing muscle strength, muscle mass, muscle quality index, and body mass index.

**Results:**

A total of 238 individuals were evaluated (119 in each group), with a mean age of 49.3 ± 14.4 years. The prevalence of sarcopenia was 18.5%, and 66.7% exhibited low muscle quality. Underweight was observed in 6.3% of the sample, while 41.4% were overweight. There were no differences in rates of underweight, low muscle mass, low muscle strength, sarcopenia, and low muscle quality according to PTH status (p > 0.05).

**Conclusion:**

Patients undergoing HD with moderate to severe hyperparathyroidism (PTH ≥ 600 pg/mL) did not show higher nutritional impairment or sarcopenia compared to patients with PTH levels within the recommended range (200–599 pg/mL).

## Introduction

In chronic kidney disease (CKD), increased parathyroid hormone (PTH) production is secondary to deteriorating renal function. Hypocalcemia, suppression of 1,25-dihydroxyvitamin D synthesis, and hyperphosphatemia contribute to secondary hyperparathyroidism, which is a common complication in hemodialysis (HD) patients^1^.

PTH is essential for maintaining calcium homeostasis through its actions in bone remodeling regulation. Its well-known catabolic and anabolic effects on the skeleton include promoting bone formation and resorption through direct effects on osteoblasts and indirect actions on osteoclasts. Bone and skeletal muscle interact anatomically and biochemically, forming a single functional system crucial for whole-body physiology. Given the functional linkage between muscle and bone, it is also important to investigate the effects of increased PTH levels on muscle tissue^2^.

Some studies have indicated an increase in resting energy expenditure and muscle loss in severe cases of hyperparathyroidism^3^. It has been demonstrated that elevated PTH levels are associated with weight loss over a 12-month period in patients receiving maintenance HD^3^. Few studies have examined the potential effect of PTH levels on nutritional status and sarcopenia in patients with CKD undergoing renal replacement therapy (RRT). An investigation involving a sample of older women without kidney disease reported that secondary hyperparathyroidism increased the risk of sarcopenia^4^. In this context, the aim of this study was to analyze the status of sarcopenia and nutritional aspects according to PTH levels in patients undergoing HD.

## Methods

This cross-sectional study enrolled consecutive patients from three HD outpatient clinics in Northeast Brazil (two public and one private). Patients were recruited by convenience sample. Inclusion criteria were having undergone HD for at least 6 months and being over 20 years old, regardless of sex. Exclusion criteria comprised previous parathyroidectomy, limb amputation, and inability to undergo nutritional and physical assessments. This study was approved by the Institutional Research Ethics Committee of the University Hospital of the *Universidade Federal de Pernambuco*. All patients signed written informed consent.

**Figure 1:**
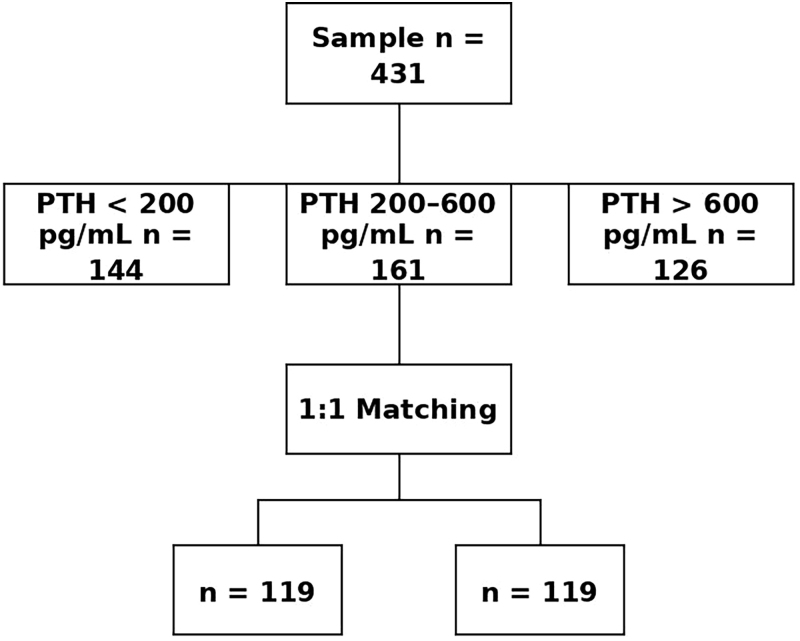
Study flowchart.

A total of 431 patients were screened for eligibility (Figure 1) and stratified into three groups based on their PTH levels: i) < 200 pg/mL; ii) 200–599 pg/mL; and iii) ≥ 600 pg/mL^5^. According to the Kidney Disease: Improving Global Outcomes (KDIGO) guidelines^6^ for patients undergoing HD, the recommended PTH level ranges from 2 to 9 times the upper limit of normal. This range corresponds to the PTH levels observed in group 2 of this study. Group 3 consisted of patients with moderate to severe hyperparathyroidism. Group 1 was excluded from the analysis due to its older age and higher likelihood of presenting chronic diseases, sarcopenia, and malnutrition^5,7^. To minimize potential biases resulting from baseline characteristics, groups 2 and 3 were matched for age, sex, and presence of diabetes, with group 3 serving as the reference group. This matching resulted in 119 patients in each group, who were included in the analyses. All patients received 4-hour HD sessions 2–3 times per week. PTH levels were measured using electrochemiluminescence and obtained from medical records, with values from the month of data collection considered for analysis.

### Sarcopenia, Muscle Quality, and Gait Speed Evaluation

Sarcopenia diagnosis was established following the European Working Group on Sarcopenia in Older People 2 (EWGSOP2) criteria, which define sarcopenia as reduced muscle strength combined with reduced muscle mass^8^.

Muscle strength was assessed using handgrip strength (HGS), measured with a JAMAR^®^ digital dynamometer. Participants remained seated with hips and knees flexed at 90º, shoulders in a neutral position, elbows flexed at 90º, and forearms in semi-pronation, without radial or ulnar deviation, following techniques established by the American Society of Hand Therapists^9^. The dynamometer grip was individually adjusted based on each participant’s hand size, ensuring that the dynamometer’s handle remained positioned over the proximal phalanges of the index, middle, and ring fingers. During the test, the arm remained stationary, with only the interphalangeal and metacarpophalangeal joints flexing. Participants received prior instructions for the procedure. Tests were performed three times on the dominant hand, with a 15-second interval between attempts to prevent fatigue. Force was applied for 5 seconds in each trial, and the highest value recorded (kg/f) was considered. Classification was based on cutoff points established by the EWGSOP2^8^.

Appendicular skeletal muscle mass (ASM) was calculated using the equation proposed by Sergi et al.^10^: ASM = (0.227 * resistance index [RI]) + (0.064 * reactance [Xc]) + (0.095 * weight [P]) + (1.384 * sex) – 3.964. Resistance and reactance measurements were obtained using bioelectrical impedance analysis (BIA) with a portable Biodynamics Model 310 device, applying a current of 800 μA at a single frequency of 50 kHz. Measurements were taken 60 minutes after the HD session, following a standardized protocol^11^. From Sergi’s equation results, ASM was normalized to height^2^ to obtain the ASM index (ASMI). Low muscle mass was determined using the Brazilian cutoff values for ASMI, which are ≤ 7.7 kg/m^2^ in men and ≤ 5.62 kg/m^2^ in women^12^.

The 4-meter gait speed (GS) test was conducted according to the model proposed by the International Academy on Nutrition and Aging (IANA)^13^. Participants were instructed to walk at their usual pace on a flat surface. The test began when the participant started walking, and the time taken to complete the entire distance was recorded using a stopwatch. The assessment was performed twice, with the faster time used as the reference. GS was classified as slow if < 0.8 m/s^8^. Muscle quality was measured using the muscle quality index (MQI), which assesses the ratio between HGS and ASM. The cutoff values proposed by Lopes et al.^14^ were used, with MQI < 3.2 indicating low muscle quality.

### Nutritional Evaluation and Body Composition

Body weight (kg) and height (m) were measured to calculate body mass index (BMI, in kg/m^2^), classified in accordance with the World Health Organization guidelines^15^. Body fat percentage (%BF) was determined using BIA with equations preprogrammed by the device manufacturers^11^. Values of %BF greater than 40% for women and greater than 30% for men were considered elevated^16^.

### Sociodemographic and Clinical Data

Data on age, sex, time on HD, and comorbidities (e.g., systemic arterial hypertension and type 2 diabetes mellitus) were obtained through interviews with patients.

### Statistics

Data were analyzed using the Statistical Package for the Social Sciences (SPSS), version 13. Descriptive statistical analysis included the calculation of frequencies, means ± standard deviations, or medians and interquartile ranges (IQR), depending on the normality of the variable, assessed by the Kolmogorov-Smirnov test. Pearson’s χ^2^ test was used to investigate associations between PTH strata and demographic, clinical, and nutritional variables. The independent Student’s t-test or the Mann-Whitney U test was employed to compare means or medians, respectively. Associations were considered significant in the final model if p < 0.05 after adjustments.

## Results

A total of 431 patients were initially recruited (Figure 1). Of these, 144 were excluded due to having PTH levels < 200 pg/mL and being older (53.1 ± 16.2 years; p = 0.012) compared to the other groups (PTH 200–599 pg/mL and PTH ≥ 600 pg/mL). In addition, they showed a higher prevalence of sarcopenia (p = 0.043) and lower mean values for both the GS test (p = 0.045) and ASMI (p = 0.031).

The final sample included 238 individuals, with a mean age of 49.3 ± 14.4 years. There was a higher proportion of men (56.7%) and adults (76.1%), with 69.3% of patients having systemic arterial hypertension and 35.3% having diabetes mellitus. PTH-stratified groups were similar in these characteristics (Table 1). The mean PTH levels in groups 2 (PTH 200–599 pg/mL) and 3 (PTH ≥ 600 pg/mL) were 348.7 ± 114.9 pg/mL and 1138.2 ± 639.5 pg/mL, respectively. Sarcopenia occurred in 18.5% of participants, while 66.7% exhibited low muscle quality (MQI). Underweight was observed in 6.3% of patients, and 41.4% were overweight. There were no significant differences in the rates of underweight, muscle mass, muscle strength, sarcopenia, and muscle quality based on PTH status (p > 0.05) (Table 2). In the comparative analysis of mean or median values according to PTH status, patients with moderate to severe hyperparathyroidism had a higher median dialysis time (p < 0.001) (Table 2).

**Table 1:** Demographic, clinical, and nutritional characteristics according to parathyroid hormone levels in patients undergoing hemodialysis (n = 238)

Variables	Overall	PTH (n = 238)		*P*
		200–599 pg/mL(n = 119)	≥ 600 pg/mL(n = 119)	
**Sex**				0.239
Male	135 (56.7)	72 (53.3)	63 (46.7)	
Female	103 (43.3)	47 (45.6)	54 (54.4)	
**Age classification**				0.288
Adults	181 (76.1)	87 (48.1)	94 (51.9)	
Older adults	57 (23.9)	32 (56.1)	25 (43.9)	
**Systemic arterial hypertension**				0.482
Yes	165 (69.3)	85 (51.5)	80 (48.5)	
No	73 (30.7)	34 (46.6)	39 (53.4)	
**Type 2 diabetes mellitus**				0.175
Yes	84 (35.3)	47 (56.0)	37 (44.0)	
No	154 (64.7)	72 (46.8)	80 (53.2)	
**ASMI**				0.968
Low	64 (27.5)	32 (50.0)	32 (50.0)	
Normal	169 (72.5)	84 (49.7)	85 (50.3)	
**HGS**				0.992
Yes	136 (57.1)	68 (50.0)	68 (50.0)	
No	102 (42.9)	51 (50.0)	51 (50.0)	
**GS**				0.983
< 0.8m/s	57 (24.6)	29 (50.9)	28 (49.1)	
> 0.8m/s	175 (75.4)	88 (50.3)	87 (49.7)	
**Sarcopenia**				0.381
Yes	190 (81.5)	92 (48.4)	98 (51.6)	
No	43 (18.5)	24 (55.8)	19 (44.2)	
**Severity of sarcopenia**				0.328
Sarcopenia	29 (67.5)	15 (51.7)	14 (49.3)	
Severe sarcopenia	14 (32.5)	9 (64.3)	5 (37.5)	
**MQI**				0.261
Low	152 (66.7)	70 (46.1)	82 (53.9)	
Normal	76 (33.3)	41 (53.9)	35 (46.1)	
**BMI**				0.180
Underweight	15 (6.3)	11 (73.3)	4 (26.7)	
Normal range	124 (52.6)	60 (48.4)	64 (51.6)	
Excess weight	98 (41.4)	48 (49.0)	50 (51.0)	
**%BF**				0.190
Normal	153 (64.6)	72 (47.1)	81 (52.9)	
High	84 (35.4)	47 (56.0)	37 (44.0	

Abbreviations – ASMI: Appendicular skeletal muscle mass index; %BF: Body fat percentage; BMI: Body mass index; GS: Gait speed; HGS: Handgrip strength; MQI: Muscle quality index; PTH: Parathyroid hormone.

**Table 2:** Comparative analysis of age, hemodialysis duration, nutritional variables, and creatinine according to parathyroid hormone levels in patients undergoing hemodialysis (n = 238)

Variables	Mean ± SD or Median (IQR)	PTH (n = 238)		*P*
		200–599 pg/mL(n = 119)	≥ 600 pg/mL(n = 119)	
Age (years)	49.3 ± 14.4	51.1 ± 14.7	47.5 ± 13.8	0.156
Duration of HD (years)	3.3 (1.6–6.6)	2.5 (1.2–5.4)	4.5 (2.3–7.4)	< 0.001
Height (m)	1.6 ± 0.8	1.8 ± 0.1	1.6 ± 0.1	0.399
BMI (kg/m^2^)	24.2 ± 4.0	24.3 ± 4.3	24.3 ± 3.7	0.995
ASMI (kg/m^2^)	7.9 ± 0.9	7.9 ± 0.9	7.9 ± 0.8	0.741
HGS (kg/f)	21.4 ± 9.1	21.8 ± 8.4	21.2 ± 9.8	0.620
GS (m/s)	1.0 ± 0.3	1.0 ± 0.3	1.0 ± 0.3	0.645
MQI	1.3 ± 0.4	2.8 ± 1.1	2.8 ± 1.3	0.670
%BF	32.0 ± 9.9	31.7 ± 10.3	32.3 ± 9.5	0.678

Abbreviations – ASMI: Appendicular skeletal muscle mass index; %BF: Body fat percentage; BMI: Body mass index; GS: Gait speed; HD: Hemodialysis; HGS: Handgrip strength; IQR: Interquartile range; MQI: Muscle quality index; PTH: Parathyroid hormone; SD: Standard deviation.

## Discussion

The findings of this study suggest that PTH levels and moderate to severe secondary hyperparathyroidism do not increase the risk of sarcopenia and muscle impairment. Similar results for BMI, muscle mass, body fat percentage, sarcopenia, and muscle quality were observed in both groups of patients (PTH 200–599 pg/mL and PTH ≥ 600 pg/mL).

Studies on the relationship between nutritional status and PTH levels are relatively scarce, but some findings point in the opposite direction, showing that elevated PTH levels can affect nutritional parameters. Epidemiological data from 42,319 patients on maintenance HD revealed an association between increased PTH levels and weight loss over a 12-month period^4^. A recent investigation also showed worsening nutritional impairment among patients on maintenance HD with severe hyperparathyroidism, despite stable dietary protein intake^3^. Another investigation demonstrated significant nutritional impairment among patients with severe hyperparathyroidism, which improved considerably after parathyroidectomy^17^. In patients undergoing peritoneal dialysis, the geriatric nutritional risk index (GNRI) was negatively correlated with PTH levels^18^.

The underlying mechanisms of this relationship were explored in an animal model of renal insufficiency. Mice that underwent 5/6 nephrectomy, resulting in elevated PTH levels, exhibited cachexia, reduced body weight, and increased energy expenditure^19^.

The lack of association between nutritional and sarcopenia parameters and PTH status revealed in our investigation may be related to the robust criteria adopted to control for confounding variables in this assessment: (1) matching characteristics that determine sarcopenia (age, sex, presence of diabetes mellitus)^20^; and (2) excluding the group of patients with PTH levels < 200 pg/mL, who were older and might have higher rates of sarcopenia. It has been shown that PTH levels increase with age and are about 30% higher in the older individuals compared to younger individuals. This increase in geriatrics is attributed to decreased calcium absorption efficiency and lower levels of 25(OH)D^21^. Therefore, it is important to consider the effects of confounding variables in the association between PTH and nutritional status.

It should be noted that PTH levels were not stratified into moderate hyperparathyroidism (PTH ≥ 600 pg/mL and < 1500 pg/mL) and severe hyperparathyroidism (PTH > 1500 pg/mL), as these patients were included in a single group (PTH ≥ 600 pg/mL). It is possible that only the most severe hyperparathyroidism status affects nutritional parameters. Despite this, we emphasize that this study is the first to investigate sarcopenia in CKD patients undergoing HD in relation to PTH levels, potentially providing initial data for future investigations.

Few studies corroborate our results, but one cannot ignore publication bias when addressing this topic, with a higher likelihood of publication of studies reporting positive results. Publication bias has been documented in the literature for decades, and its origins and consequences have been widely debated, with evidence suggesting that this bias is increasing. The highly competitive environment for funding and career advancement may encourage researchers to submit predominantly positive results for publication, knowing they are more likely to be considered by editors, reviewed more favorably by peers, and, once published, more likely to be cited^22,23^.

Although the relationship between sarcopenia and muscle impairment was not detected in our results, it is important to systematically evaluate these parameters in clinical practice, providing opportunities for intervention strategies when nutritional deviations are identified, thereby mitigating related complications and adverse outcomes^24,25^.

In interpreting our results, several aspects should be considered. First, our investigation did not assess the level of physical activity, a parameter that can directly affect muscle mass and sarcopenia status. Therefore, we do not know whether one group was more physically active than the other. However, we evaluated the GS test, which can reflect individuals’ functionality and physical condition, and this assessment showed equivalence between groups. Second, we considered only the result of a single PTH test. Third, the cross-sectional design precludes causal inferences, reinforcing the need for longitudinal studies to prospectively assess changes in nutritional parameters over the course of PTH level progression. Future investigations with randomized samples and adjusted confounding factors should be undertaken to achieve more robust results. Additionally, the strengths of the present study included the matching of patient data across different degrees of hyperparathyroidism and the analysis of multiple nutritional parameters associated with sarcopenia and nutritional status.

In conclusion, patients undergoing HD with moderate to severe hyperparathyroidism (PTH ≥ 600 pg/mL) did not show a higher risk of nutritional impairment and sarcopenia compared to patients with PTH levels within the recommended range (200–599 pg/mL). Further longitudinal studies are warranted to achieve more precise insights into this relationship.

## Data Availability

The datasets generated and/or analyzed during the current study are available from the corresponding author upon reasonable request.
