# Supplementary material for: Relationship between parathyroid hormone levels and sarcopenia and nutritional status in patients undergoing hemodialysis
Source: J Bras Nefrol. 2026 Aug 7;48(3):e20250083. doi: 10.1590/2175-8239-JBN-2025-0083en (PMC13450942; doi:10.1590/2175-8239-JBN-2025-0083en)
Supplement: Translation

# Translation: Relação entre níveis de paratormônio e status de sarcopenia e estado nutricional em pacientes em hemodiálise

**DOI:** 10.1590/2175-8239-JBN-2025-0083pt

**Authors:** Claudia Campello Leal, Dejane de Almeida Melo, Alcione Miranda dos Santos, Maria Conceição Chaves de Lemos, Pedro Israel Lira, Claudia Porto Sabino Pinho

**Affiliations:** 1 Universidade Federal de Pernambuco, Departamento de Nutrição, Recife, PE, Brasil.; 2 Instituto de Gestão Estratégica de Saúde do Distrito Federal, Hospital de Base, Brasília, DF, Brasil.; 3 Universidade Federal do Maranhão, Recife, PE, Brasil.; 4 Universidade Federal de Pernambuco, Empresa Brasileira de Serviços Hospitalares, Hospital das Clínicas, Recife, PE, Brasil.

**Keywords:** Hormônio Paratireóideo, Insuficiência Renal Crônica, Diálise Renal, Sarcopenia, Desnutrição

## Abstract

**Introdução:**

Ainda não está estabelecido o potencial efeito de elevados níveis de paratormônio (PTH) no estado nutricional e no *status* de sarcopenia em pacientes com doença renal crônica (DRC) submetidos à (HD).

**Objetivo:**

Analisar o *status* de sarcopenia e aspectos nutricionais segundo os níveis de PTH em pacientes em HD.

**Métodos:**

Estudo transversal que avaliou pacientes regulados em três clínicas de HD no Nordeste do Brasil. Os pacientes foram estratificados segundo os níveis de PTH: (1) 0–199 pg/mL, (2) 200–599 pg/mL e (3) ≥ 600 pg/mL. O grupo 1 foi excluído por conter os pacientes com maior idade, e os grupos 2 e 3 foram pareados segundo idade, sexo e presença de diabetes. A sarcopenia foi estabelecida segundo critério europeu, sendo avaliados: força muscular, massa muscular, índice de qualidade muscular e índice de massa corporal.

**Resultados:**

Foram avaliados 238 indivíduos (119 em cada grupo), com média de idade de 49,3 ± 14,4 anos. A prevalência de sarcopenia foi de 18,5%, e 66,7% exibiram baixa qualidade muscular. A desnutrição foi observada em 6,3% da amostra, e 41,4% tinham excesso de peso. Não houve diferença nas taxas de desnutrição, baixa massa muscular, baixa força muscular, sarcopenia e baixa qualidade muscular segundo o *status* de PTH (p > 0,05).

**Conclusão:**

pacientes em HD com hiperparatireoidismo moderado e grave (PTH ≥ 600 pg/mL) não apresentaram maior comprometimento nutricional ou sarcopenia em comparação com pacientes com níveis de PTH dentro da faixa recomendada (200–599 pg/mL).

## Introdução

Na doença renal crônica, o aumento da produção de paratormônio (PTH) é secundário ao declínio da função renal. A hipocalcemia, supressão da síntese de 1,25-di-hidroxivitamina D e a hiperfosfatemia contribuem para o hiperparatireoidismo secundário, sendo essa uma complicação comum em pacientes em hemodiálise (HD)^1^.

O PTH é fundamental para a manutenção da homeostase do cálcio, por meio de suas ações na regulação da remodelação óssea, e as ações catabólicas e anabólicas que exerce sobre o esqueleto são bem conhecidas e relatadas. Este hormônio pode promover a formação e a reabsorção ósseas por meio de efeitos diretos sobre os osteoblastos e de ações indiretas sobre os osteoclastos. Osso e músculo esquelético interagem anatomicamente e bioquimicamente entre si, sendo considerados um sistema único funcional, cuja relação é essencial para a fisiologia do corpo inteiro. Como o músculo está funcionalmente ligado ao osso, também é importante investigar os efeitos do aumento do PTH sobre o tecido muscular^2^.

Alguns estudos também sugeriram um aumento no gasto energético de repouso e na perda muscular em situações graves de hiperparatireoidismo, tendo sido demonstrada uma relação entre o aumento do nível de PTH e a perda de peso em 12 meses em pacientes recebendo hemodiálise de manutenção^3^. Poucos estudos examinaram o potencial efeito dos níveis de PTH no estado nutricional e no *status* de sarcopenia em pacientes com doença renal crônica (DRC) em terapia de reposição renal (TRR). Uma investigação com uma amostra de mulheres idosas livres de doença renal reportou que o hiperparatireoidismo secundário aumentou o risco de sarcopenia^4^. Nesse contexto, o objetivo deste estudo foi analisar o *status* de sarcopenia e aspectos nutricionais segundo os níveis de PTH em pacientes em HD.

## Métodos

Estudo transversal envolvendo pacientes consecutivos de três ambulatórios de HD no Nordeste do Brasil (dois públicos e um privado). Os pacientes foram recrutados por conveniência. Os critérios de inclusão foram: ter sido submetido à HD por pelo menos 6 meses e ter mais de 20 anos de idade, independentemente do sexo. Os critérios de exclusão incluíram paratireoidectomia prévia, amputação de membros e incapacidade de se submeter a avaliações nutricionais e físicas. Este estudo foi aprovado pelo Comitê de Ética em Pesquisa do Hospital das Clínicas da Universidade Federal de Pernambuco. Todos os pacientes assinaram o Termo de Consentimento Livre e Esclarecido (TCLE).

Um total de 431 pacientes foi selecionado a partir dos critérios de elegibilidade (Figura 1) e estratificado em três grupos com base em seus níveis de PTH: i) < 200 pg/mL; ii) 200–599 pg/mL; iii) ≥ 600 pg/mL^5^. De acordo com as diretrizes da *Kidney Disease: Improving Global Outcomes* (KDIGO)^6^ para pacientes submetidos à HD, o nível de PTH recomendado varia de 2 a 9 vezes o limite superior do normal. Essa faixa corresponde aos níveis de PTH observados no grupo 2 deste estudo. O grupo 3 consistiu em pacientes com hiperparatireoidismo moderado a grave. O grupo 1 foi excluído da análise devido à sua idade avançada e à maior probabilidade de apresentar doenças crônicas, sarcopenia e desnutrição^5,7^. Para minimizar os potenciais vieses decorrentes das características basais, os grupos 2 e 3 foram pareados por idade, sexo e presença de diabetes, com o grupo 3 servindo como referência. Essa comparação resultou em 119 pacientes em cada grupo, que foram incluídos nas análises. Todos os pacientes receberam sessões de HD de 4 horas, 2 a 3 vezes por semana. Os níveis de PTH foram medidos por eletroquimioluminescência e obtidos de prontuários médicos, sendo considerados para análise os valores referentes ao mês da coleta.

**Figura 1:** Fluxograma da amostra.

### Avaliação da Sarcopenia, Qualidade Muscular e Velocidade de Marcha

O diagnóstico da sarcopenia foi estabelecido pelos critérios do EWGSOP2, que estabelece como sarcopênico o indivíduo que possui força muscular reduzida associada à massa muscular reduzida^8^.

A força muscular (FM) foi avaliada a partir da força de preensão palmar (FPP), mensurada com o uso do dinamômetro digital JAMAR^®^. O participante permaneceu sentado com os quadris e os joelhos fletidos a 90º, o ombro aduzido em posição neutra, o cotovelo fletido a 90º e o antebraço em semipronação, sem que houvesse desvio radial ou ulnar, segundo técnicas estabelecidas pela *American Society of Hand Therapists* ^9^. A pegada no dinamômetro foi ajustada individualmente de acordo com o tamanho das mãos de cada indivíduo, de forma que a haste mais próxima do corpo do dinamômetro permanecesse posicionada sobre as segundas falanges dos dedos indicador, médio e anelar. Durante a preensão manual, o braço permaneceu imóvel, havendo somente a flexão das articulações interfalangeanas e metacarpofalangeanas. Os participantes receberam instruções prévias para a realização do procedimento. Os testes foram realizados três vezes, na mão dominante, com intervalo de 15 segundos entre cada tentativa, a fim de evitar fadiga durante o teste. A força foi aplicada durante 5 segundos em cada tentativa, sendo considerada a medida de maior valor, com os resultados registrados em kg/f. Para a classificação, foram considerados os pontos de corte estabelecidos pelo Consenso Europeu^8^.

A massa muscular apendicular esquelética (MMAE) foi obtida a partir da equação de Sergi et al.^10^: ASM = (0,227* índice de resistência [RI]) + (0,064* reatância [Xc]) + (0,095* peso [P]) + (1,384* sexo) – 3,964. A medida de resistência e reatância foi obtida a partir de bioimpedância elétrica (BIA), utilizando-se o equipamento portátil da marca *Biodynamics*, modelo 310, que aplica uma corrente de 800 μA e frequência simples de 50 kHz. As medições foram realizadas 60 minutos após a sessão de HD, utilizando-se um protocolo previamente estabelecido^11^. A partir do resultado da equação de Sergi et al.^10^, calculou-se o índice de massa muscular esquelética apendicular (IMMAE), utilizando a fórmula: MMAE/altura^2,8^. O IMMAE foi classificado segundo o ponto de corte sugerido para a população brasileira, em que valores ≤ 7,7 kg/m^2^ em homens e ≤ 5,62 kg/m^2^ em mulheres foram considerados indicativos de baixa massa muscular^12^.

O teste de velocidade de marcha (TVM) foi aplicado segundo modelo proposto pela *International Academy on Nutrition and Aging* (IANA)^13^, sendo executado em um percurso de 4 metros, orientando-se o participante a andar no seu passo habitual, em local com piso plano. Ao iniciar o teste, cronometrou-se o tempo gasto para a realização completa do trajeto. A avaliação foi realizada em duplicata, sendo adotado como referência o percurso realizado em menor período de tempo. A marcha foi considerada lenta quando a velocidade foi < 0,8 m/s^8^. A qualidade muscular foi mensurada considerando-se o índice de qualidade muscular (IQM), que utiliza a razão da força muscular por unidade de massa muscular apendicular esquelética (FPP/MMAE), sendo considerados os valores de corte propostos por Lopes et al.^14^, que estabelecem como baixa qualidade muscular os valores de IQM < 3,2.

### Avaliação Nutricional e Composição Corporal

Foi avaliado o índice de massa corporal (IMC), considerando-se o quociente entre o peso (kg) dividido pela altura (m) ao quadrado e a classificação estabelecida pela Organização Mundial da Saúde^15^. O percentual de gordura foi determinado por BIA diretamente por meio de equações já programadas pelos fabricantes do aparelho^11^. Os valores de percentual de gordura corporal (%GC) superiores a 40% para mulheres e 30% para homens foram considerados elevados^16^.

### Avaliação Demográfica e Clínica

Foram considerados os dados de idade, sexo, presença de hipertensão e diabetes e tempo de HD, obtidos a partir de entrevista com o paciente.

### Análise Estatística

Os dados foram analisados no software *Statistical Package for the Social Sciences* (SPSS) – versão 13. A análise estatística descritiva dos dados foi realizada por meio do cálculo das frequências, médias e desvios padrão ou medianas e intervalo interquartílico, conforme o padrão de normalidade da variável, avaliado pelo teste de *Kolmogorov-Smirnov*. O teste qui-quadrado de *Pearson* foi empregado para investigar a associação entre os estratos de PTH e as variáveis demográficas, clínicas e nutricionais. O teste t de *Student* ou U de *Mann-Whitney* foi adotado para a comparação de médias ou medianas, respectivamente. Para a aceitação das associações investigadas no modelo final, após os ajustes, foi adotado o valor de p < 0,05.

## Resultados

O fluxograma do estudo está apresentado na Figura 1, com um total de 431 pacientes recrutados, dos quais 144 tinham níveis de PTH < 200 pg/mL, sendo excluídos das análises por apresentarem maior idade (53,1 ± 16,2 anos; p = 0,012) quando comparados aos outros dois grupos (PTH 200–599 pg/mL e PTH ≥ 600 pg/mL). Além disso, apresentaram maior prevalência de sarcopenia (p = 0,043) e menores valores médios tanto para o teste de VM (p = 0,045) quanto para o IMMAE (p = 0,031).

Foram avaliados 238 indivíduos, cuja média de idade foi 49,3±14,4 anos. Verificou-se maior proporção de homens (56,7%) e adultos (76,1%), sendo evidenciado que 69,3% dos pacientes eram portadores de HAS e 35,3% de DM. Os grupos estratificados segundo os níveis de PTH foram equivalentes em relação a essas características (Tabela 1). As médias dos níveis de PTH no grupo 2 (PTH 200–599 pg/mL) e no grupo 3 (PTH ≥ 600 pg/mL) foram 348,7±114,9 pg/mL e 1138,2±639,5 pg/mL, respectivamente. A prevalência de sarcopenia foi de 18,5%, e 66,7% dos pacientes apresentaram baixa qualidade muscular. A desnutrição foi observada em 6,3% da amostra e 41,4% tinham excesso de peso. Não houve diferença nas taxas de desnutrição, massa muscular, força muscular, sarcopenia e qualidade muscular segundo o *status* de PTH (p > 0,05) (Tabela 2). Na análise comparativa dos valores médios ou medianos, segundo o *status* de PTH, observou-se que pacientes com hiperparatireoidismo moderado e grave apresentaram maior mediana do tempo de HD (p < 0,001) (Tabela 2).

**Tabela 1:** Características demográficas, clínicas e nutricionais segundo os níveis de paratormônio em pacientes em hemodiálise

Variável	Total	PTH (n = 238)		Valor de p
		200–599 pg/mL(n = 119)	≥ 600pg/mL(n = 119)	
**Sexo**				0,239
Masculino	135 (56,7)	72 (53,3)	63 (46,7)	
Feminino	103 (43,3)	47 (45,6)	54 (54,4)	
**Faixa etária**				0,288
Adulto	181 (76,1)	87 (48,1)	94 (51,9)	
Idoso	57 (23,9)	32 (56,1)	25 (43,9)	
**HAS**				0,482
Sim	165 (69,3)	85 (51,5)	80 (48,5)	
Não	73 (30,7)	34 (46,6)	39 (53,4)	
**DM**				0,175
Sim	84 (35,3)	47 (56,0)	37 (44,0)	
Não	154 (64,7)	72 (46,8)	80 (53,2)	
**IMMAE**				0,968
Baixo	64 (27,5)	32 (50,0)	32 (50,0)	
Normal	169 (72,5)	84 (49,7)	85 (50,3)	
**Força Muscular**				0,992
Baixa	136 (57,1)	68 (50,0)	68 (50,0)	
Normal	102 (42,9)	51 (50,0)	51 (50,0)	
**VM**				0,983
< 0,8 m/s	57 (24,6)	29 (50,9)	28 (49,1)	
> 0,8 m/s	175 (75,4)	88 (50,3)	87 (49,7)	
**Sarcopenia**				0,381
Não	190 (81,5)	92 (48,4)	98 (51,6)	
Sim	43 (18,5)	24 (55,8)	19 (44,2)	
**Grau de sarcopenia**				0,328
Sarcopenia	29 (67,5)	15 (51,7)	14 (49,3)	
Sarcopenia grave	14 (32,5)	9 (64,3)	5 (37,5)	
**Qualidade Muscular**				0,261
Baixa	152 (66,7)	70 (46,1)	82 (53,9)	
Normal	76 (33,3)	41 (53,9)	35 (46,1)	
**IMC**				0,180
Desnutrição	15 (6,3)	11 (73,3)	4 (26,7)	
Eutrofia	124 (52,6)	60 (48,4)	64 (51,6)	
Excesso de Peso	98 (41,4)	48 (49,0)	50 (51,0)	
**%Gordura**				0,190
Normal	153 (64,6)	72 (47,1)	81 (52,9)	
Elevado	84 (35,4)	47 (56,0)	37 (44,0)	

Abreviaturas – PTH: Paratormônio; HAS: Hipertensão arterial sistêmica, DM: Diabetes mellitus; IMMAE: Índice de massa muscular apendicular esquelética.

**Tabela 2:** Análise comparativa da idade, tempo de hemodiálise, variáveis nutricionais e creatinina segundo os níveis de paratormônio em pacientes em hemodiálise

Variáveis	Média (DP) ou Mediana (IIQ)	PTH (n = 238)		Valor de p
		200–599 pg/mL(n = 119)	≥ 600 pg/mL(n = 119)	
Idade (anos)	49,3 ± 14,4	51,1 ± 14,7	47,5 ± 13,8	0,156
Tempo de HD (anos)	3,3 (1,6–6,6)	2,5 (1,2–5,4)	4,5 (2,3–7,4)	< 0,001
Altura (m)	1,6 ± 0,8	1,8 ± 0,1	1,6 ± 0,1	0,399
IMC (kg/m^2^)	24,2 ± 4,0	24,3 ± 4,3	24,3 ± 3,7	0,995
IMMAE (kg/m^2^)	7,9 ± 0,9	7,9 ± 0,9	7,9 ± 0,8	0,741
FPP (kg/f)	21,4 ± 9,1	21,8 ± 8,4	21,2 ± 9,8	0,620
VM (m/s)	1,0 ± 0,3	1,0 ± 0,3	1,0 ± 0,3	0,645
IQM	1,3 ± 0,4	2,8 ± 1,1	2,8 ± 1,3	0,670
%Gordura	32,0 ± 9,9	31,7 ± 10,3	32,3 ± 9,5	0,678

Abreviaturas – PTH: Paratormônio; IMC: Índice de massa corporal; IMMAE: Índice de massa muscular apendicular esquelética; FPP: Força de preensão palmar; IQM: Índice de qualidade muscular; VM: Velocidade de marcha.

## Discussão

Os achados desse estudo refutam a hipótese de que os níveis de PTH e o hiperparatireoidismo secundário moderado e grave aumentam o risco de sarcopenia e comprometimento muscular. Resultados equivalentes para IMC, massa muscular, percentual de gordura, sarcopenia e qualidade muscular foram evidenciados nos dois grupos de pacientes avaliados (PTH 200–599 pg/mL e PTH ≥ 600 pg/mL).

Não há muitos estudos sobre a relação entre o estado nutricional e os níveis de PTH, mas alguns resultados apontam para a direção oposta, demonstrando que níveis elevados desse hormônio podem afetar parâmetros nutricionais. Dados epidemiológicos de 42.319 pacientes em HD de manutenção revelaram uma associação entre o aumento do nível de PTH e a perda de peso em 12 meses^4^. Uma recente investigação também revelou piora do comprometimento nutricional entre pacientes em HD de manutenção com hiperparatireoidismo grave, apesar da ingestão estável de proteínas na dieta^3^. Outra investigação demonstrou comprometimento nutricional substancial entre pacientes com hiperparatireoidismo grave, que melhorou consideravelmente após a paratireoidectomia^17^. Em pacientes em diálise peritoneal, o índice de risco nutricional geriátrico (IRNG) foi negativamente correlacionado com os níveis de PTH^18^.

Os mecanismos subjacentes a essa relação foram explorados em um modelo animal de insuficiência renal. Os camundongos submetidos à nefrectomia 5/6, resultando em níveis elevados de PTH, apresentaram caquexia, redução do peso corporal e aumento do gasto energético^19^.

A ausência de associação entre os parâmetros nutricionais e os de sarcopenia com o *status* de PTH revelada em nossa investigação pode estar relacionada ao fato de termos adotado critérios robustos para controlar variáveis de confusão na avaliação dessa associação: (1) pareamento de características determinantes de sarcopenia (idade, sexo e presença de DM^20^); e (2) exclusão do grupo de pacientes com níveis de PTH < 200 pg/mL, os quais tinham maior idade, fator que pode afetar as taxas de sarcopenia. Já foi demonstrado que os níveis de PTH aumentam com a progressão da idade e são cerca de 30% maiores em idosos do que em jovens. Esse aumento em idosos é atribuído à diminuição da eficiência de absorção de cálcio e à redução dos níveis de 25(OH)D^21^. Sendo assim, devem ser considerados os efeitos de variáveis de confusão nos resultados de associação entre PTH e *status* nutricional.

Cabe destacar que não estratificamos os níveis de PTH em hiperparatireoidismo moderado (PTH ≥ 600 pg/mL e < 1500 pg/mL) e grave (PTH > 1500 pg/mL), sendo esses pacientes incluídos em um grupo único (PTH ≥ 600 pg/mL). É possível que apenas o *status* mais grave de hiperparatireoidismo afete medidas nutricionais. Apesar disso, destacamos que o presente estudo é o primeiro a investigar o *status* de sarcopenia em pacientes com DRC submetidos à HD em relação aos níveis de PTH, podendo contribuir com dados iniciais para investigações futuras.

Poucos estudos corroboram os nossos resultados, mas não se pode deixar de considerar viés de publicação na abordagem da temática em pauta, com maior probabilidade de publicação de investigações com resultados positivos. O viés de publicação é documentado na literatura há décadas, e as suas origens e consequências têm sido amplamente debatidas, havendo evidências que sugerem que este viés vem aumentando. O ambiente altamente competitivo para financiamento e promoção de carreira pode incitar os investigadores a submeterem resultados predominantemente positivos para publicação, sabendo que têm maior probabilidade de serem considerados pelos editores, revistos mais favoravelmente pelos pares e, uma vez publicados, maior probabilidade de serem citados^22,23^.

Embora a relação entre sarcopenia e comprometimento muscular não tenha sido detectada em nossos resultados, não se deve deixar de avaliar sistematicamente esses parâmetros na prática clínica, oportunizando estratégias de intervenção quando desvios nutricionais forem identificados, com o objetivo de atenuar complicações e desfechos adversos associados^24,25^.

Na interpretação dos nossos resultados, alguns aspectos devem ser considerados. Primeiro, nossa investigação não avaliou o nível de atividade física, parâmetro que pode afetar diretamente a massa muscular e o *status* de sarcopenia. Sendo assim, não se sabe se um grupo era fisicamente mais ativo que o outro. No entanto, avaliamos o teste de velocidade de marcha, aspecto que pode refletir a funcionalidade e a condição física dos indivíduos, e essa avaliação demonstrou equivalência entre os grupos. Segundo, consideramos apenas o resultado de um único exame de PTH. Terceiro, o delineamento transversal limita inferências causais, sendo necessário o desenvolvimento de estudos longitudinais que avaliem prospectivamente as mudanças nos parâmetros nutricionais no curso da evolução dos níveis de PTH. Investigações futuras com amostras randomizadas e fatores de confusão ajustados devem ser realizadas para alcançar resultados mais robustos. Os pontos fortes do presente estudo foram o pareamento de dados de pacientes com diferentes graus de hiperparatireoidismo e a análise de múltiplos parâmetros nutricionais associados à sarcopenia e ao estado nutricional.

Concluindo, pacientes em HD com hiperparatireoidismo moderado e grave (PTH ≥ 600 pg/mL) não apresentaram maior risco de comprometimento nutricional e sarcopenia em comparação com pacientes com nível de PTH dentro da faixa recomendada (200–599 pg/mL). Mais estudos com delineamento longitudinal devem ser empreendidos para alcançar resultados mais precisos sobre essa relação.

## Data Availability

Os conjuntos de dados gerados e/ou analisados durante o presente estudo estão disponíveis com o autor correspondente, mediante solicitação razoável.
